# Implications of the MDA Trial in Southern Province, Zambia, for Malaria Control and Elimination

**DOI:** 10.4269/ajtmh.19-0673

**Published:** 2020-07-02

**Authors:** Richard W. Steketee, John M. Miller, Elizabeth Chizema Kawesha

**Affiliations:** 1 PATH Malaria Control and Elimination Partnership in Africa (MACEPA), Lusaka, Zambia;; 2 National Malaria Control Centre, Zambia Ministry of Health, Chainama Hospital Grounds, Lusaka, Zambia

In this supplement, we present findings from a cluster randomized controlled trial to assess the relative effectiveness of community-wide mass drug administration (MDA) or household focal MDA (fMDA) compared with the standard of care. The trial was carried out in 60 health facility catchment areas (HFCAs) along Lake Kariba in Zambia’s Southern Province between December 2014 and February 2016. We evaluated these strategies in areas of higher (*Plasmodium falciparum* prevalence > 10%, with a mean of approximately 50%) and lower (prevalence < 10%, with a mean approximately 8%) transmission settings. Both mass treatment strategies used a long-acting antimalarial drug (dihydroartemisinin–piperaquine) that could complement the current package of standard interventions by reducing the human parasite reservoir and preventing parasitemia for up to 1 month, with the overall goal of markedly reducing overall transmission. We evaluated these interventions in a large population (approximately 330,000 people) in Southern Province, Zambia, in an attempt to maximize power across multiple transmission settings.^1^

Notably, all study arms in the trial areas received a standard of care with higher intervention coverage than typically seen nationally; these included high coverage of long-lasting insecticide-treated nets (LLINs) through a nationwide distribution program, targeted indoor residual spraying (IRS) with the highly effective pirimiphos-methyl, strengthened case management with reliable stocks of rapid diagnostic tests (RDTs) and artemisinin-based combination therapy (ACT), and expanded access to case management through community health workers (CHWs). Finally, improved surveillance, monitoring, and evaluation were supported in all trial areas to inform and evaluate the study and facilitate case investigation where feasible. Thus, the MDA and fMDA trial arms were carried out in the context of this scaled intervention package (SIP).

## SUMMARY FINDINGS

### Mass drug administration reduced malaria beyond that achieved with the SIP and worked better than fMDA.

Populations receiving MDA had consistently lower parasite prevalence,^1^ fewer incident infections in the incident cohort study population,^2^ lower confirmed malaria case incidence,^1^ and an increase in the proportion of monogenomic infections,^1,3^ compared with the SIP alone (Summary Table 1). This overall pattern was most pronounced in lower transmission settings; the point estimates of effect sizes varied but were consistently higher in the lower transmission settings than those in the higher transmission settings. Because of the overall dramatic decline in transmission and the small number of infections in all trial groups, some of the reductions in malaria outcome measures were not statistically significant in the lower transmission areas. The fMDA arm was never significantly better than the control arm statistically but consistently had point estimates that fell between the MDA arm and the control arm. The costs of MDA and fMDA per person targeted and reached were similar, but MDA was found to be more cost-effective.^4^

**Table 1:** Summary findings

		Prevalence effect size (adjusted odds ratio)		Cumulative incidence effect size (adjusted IRR)		Monthly incidence effect size, all rounds (adjusted differences in differences IRR)	
		After rounds 1 and 2	After rounds 3 and 4	After rounds 1 and 2 (rainy season)	After rounds 3 and 4 (rainy season)	After rounds 1 and 2	After rounds 3 and 4
Low transmission	MDA	0.13 (0.02–0.92)*	0.53 (0.08–3.36)	0.23 (0.03–1.82)	0.40 (0.05–3.23)	0.59 (0.44–0.78)*	0.71 (0.49–1.04)
	fMDA	0.57 (0.13–2.50)	2.60 (0.57–11.83)	1.53 (0.31–7.59)	0.66 (0.09–4.94)	0.90 (0.70–1.15)	1.08 (0.77–1.52)
High transmission	MDA	0.86 (0.25–3.04)	1.31 (0.49–3.49)	0.34 (0.13–0.89)*	0.80 (0.26–2.42)	0.92 (0.73–1.15)	1.03 (0.72–1.47)
	fMDA	1.28 (0.36–4.60)	1.13 (0.40–3.19)	0.50 (0.19–1.35)	0.96 (0.31–3.00)	1.00 (0.80–1.25)	1.20 (0.84–1.70)

fMDA = focal MDA; MDA = mass drug administration.

The benefits seen with MDA persisted for 3–4 months after the second round, with the duration of benefit longer in the lower transmission settings than that in the higher transmission settings. In initial lower transmission areas, the effect spanned nearly the entire transmission season and was observed as a greater than 70% reduction in parasite prevalence beyond what was achieved by the SIP alone. In the high-transmission areas, the effect appeared to be shorter with new blood-stage infections emerging more quickly and parasite prevalence ultimately similar across the trial groups by the end of the transmission season.

### Mass drug administration and fMDA were delivered as planned, as was the SIP.

The MDA and fMDA treatment coverage achieved in the respective trial arms were relatively high. Numerous methods of supporting and determining coverage were examined.^1,5,6^ These interventions were acceptable to the community^5^ and well-tolerated.^1^ More than 85% of households were reached by at least one campaign round, and the per-protocol evaluation identified that treatment across the four rounds was received by 59% of individuals in the MDA arm and 13% of those in the fMDA arm (treatment given only to members of the household where testing identified at least one RDT-positive household member).^6^ In sum, a substantial proportion of the population was reached by an MDA campaign. However, because of individual exclusions and challenges in finding every house and all eligible individuals, attaining even higher coverage represents a substantial challenge.

The SIP was delivered at high coverage through existing program mechanisms including the expanded CHW program that provided community case management. Among respective baseline and during trial coverages for key vector control interventions (summaries across intervention groups from Table 2 in Eisele et al.^1^):

1. Long-lasting insecticide-treated net ownership of at least one net per household increased from approximately 72% to 80%, with most nets having been replaced with new nets with fresh insecticide and recent IRS with a highly effective insecticide increased from approximately 15% to 50%.
2. Recent reported fever illness decreased among children from approximately 23–15%, and the proportion seeking care from a public or private health worker remained stable at approximately 65%.
3. The proportion of sick children seeking care from CHWs nearly doubled from 10% to 20%. Because of the deployment of CHWs, the proportion of households that were within 1.5 km of a malaria treatment provider increased from 13% to 43%.

### Malaria decreased dramatically across all study arms.

Confirmed malaria cases, infection incidence, infection prevalence, and malaria deaths were markedly reduced across the trial area in all arms of the study. Overall, parasite prevalence declined from 31.3% at baseline to 4.0% after 2 years of the trial—an 87% reduction.^1^ Summaries across intervention groups from the trial (summary of Table 4 in Eisele et al.^1^) show that in high-transmission areas in all groups combined, parasite prevalence was reduced from a baseline of 52.8–15.8% after the first year (first two rounds of MDA) and further to 6.5% after the second year (after all four rounds of MDA)—an 88% reduction after 2 years. In lower transmission settings in all groups combined, parasite prevalence was reduced from a baseline of 8.5–1.4% after the first year and remained low at 1.8% after the second year—an 81% reduction over the 2 years, with the reductions occurring dramatically in the first year. Summaries across intervention groups from the trial (summary of Table 5 in Eisele et al.^1^) show that the average rainy season monthly confirmed case incidence per 1,000 population decreased dramatically from the pre-intervention interval to the post-intervention period in both lower transmission areas (from 20.5 to 5.4 cases per 1,000) and high-transmission areas (from 60.8 to 19.4 cases per 1,000). Overall, confirmed cases in the trial area declined by 70% and increased from 1,744 at the baseline in 2014–530 in 2016. Although systematic classification of malaria as a primary cause of hospitalization or death is challenging and limits our comparisons during this trial, during comparable intervals in the trial sites, data from the routine health information system show that confirmed malaria deaths declined by 87% from 30 in 2014 to four in 2016.

### The SIP contributed to the large declines in malaria.

Although the SIP was not fundamentally different in content from the national standard that had been delivered in previous years, several additions were made by the national program that scaled up the standard package. The involved districts assured that there were no stock-outs of RDTs and first-line ACTs for case management. There was also an effort to ensure population-wide coverage of LLINs with a mass distribution campaign in August 2014, just before the first mass treatment round. Three rounds of targeted IRS with pirimiphos-methyl were conducted by the national program just before the rainy season in November each year from 2014 through 2016, reaching about 50% of the population living in the areas with the highest confirmed malaria case incidence, irrespective of mass treatment assignment.

The CHW program was expanded as part of a nationwide program seeking to deploy approximately one CHW per 850 population with the additional CHWs linked to their health facility, improving timely access to diagnosis and treatment (for malaria and other diseases) across the trial areas. The CHWs in these areas were specifically trained in the use of RDTs and ACTs at the outset of the trial. After the CHW scale-up, about 15–20% of fever patients sought treatment from a CHW instead of traveling to a health center.^1^

Quality assurance and timely reporting through the District Health Information System 2 were instituted for the information systems, and confirmed case reporting was made by all facilities and included reporting from the CHWs linked to the health facilities. When the number of confirmed cases diminished, it became feasible in many areas to investigate the individual cases with household visits and testing and treatment of other infected residents.

An important aspect of the SIP was the way in which it was coordinated and managed by the national program. This included assigning the HFCA as the primary SIP implementation unit. The HFCAs were then ranked using DHIS2 data on confirmed malaria case incidence, with the highest burden catchments receiving the most attention in achieving high SIP coverage, especially for the targeted IRS. Each year an iterative process of reprioritizing coverage of IRS and the expanding case management was conducted along with data reviews from the increased surveillance efforts.

The trial control areas that received no mass treatment campaigns but only the SIP experienced dramatic reductions in confirmed malaria cases and infection prevalence.^1^ In these control areas at baseline and after four rounds of the trial, confirmed case rates per 1,000 population decreased in lower transmission areas from 24.3 to 5.3 (a 78% reduction) and in high-transmission areas from 78.9 to 8.3 (an 89% reduction). In these same intervals and populations, child malaria parasite prevalence decreased in lower transmission areas from 9.3% to 1.4% (an 85% reduction) and in high-transmission areas from 56% to 5.0% (a 91% reduction).^1^ The reductions in control areas in malaria burden are remarkable and warrant further investigation in their own right because this trial was not specifically designed to test the contribution of the SIP.

### Importation of infections from outside the study area was not a driving force for transmission.

Reported travel outside the districts in the previous 2 weeks fluctuated seasonally and was significantly associated with risk of a parasite infection (RDT positivity). Travelers overall had 2.6 times the risk of having an infection compared with non-travelers, and individuals in lower transmission areas who traveled to higher transmission areas had 7.5 times the risk of an infection compared with their non-traveling counterparts.^7^ However, travel outside the district was reported by less than 1% of the people tested during the study. If travel patterns remained the same, there would need to be a further 98% reduction in local malaria transmission for travel-associated malaria to make up one-half of all cases. Local travel within the districts, especially travel to the shore of Lake Kariba with the lowest elevation and highest transmission in the province, may have been an important contributor to transmission; however, this was not systematically assessed.

### Observed benefits occurred in the context of growing anopheline resistance to pyrethroids^8^ but persistent parasite susceptibility to ACTs.^9^

Zambia had been deploying large numbers of pyrethroid-containing LLINs nationwide and administering IRS (mostly with pyrethroids, with some use of bendiocarb and DDT) to targeted populations consistently for more than a decade. The current trial introduced primiphos-methyl for IRS and continued the pyrethroid-containing LLINs. Bio-efficacy vector studies showed that whereas pirimiphos-methyl was highly effective against *Anopheles funestus*, pyrethroids were not.^7^ Reassuringly, monitoring of parasite susceptibility to the antimalarials used in the study showed no evidence of treatment failure, although the study was not designed to be able to draw strong conclusions about the presence or absence of treatment failure.

### Malaria elimination was not achieved.

Neither the SIP nor the addition of MDA and fMDA was sufficient to achieve local interruption of transmission as measured by zero confirmed malaria cases, although in many trial catchments, parasite prevalence dropped below 1%.^1^ Historically, this setting has been highly malarious^10^ and getting to zero cases will likely take much longer than a 2-year trial; indeed, next-generation intervention strategies and tools may be required.

## DISCUSSION

In this multiyear large-scale community trial, we established that malaria interventions—the national standard intervention package delivered in a scaled mode and compared with the addition of MDA either for the entire population or for every household with a documented current infection—were effectively delivered by the National Malaria Elimination Programme (NMEP). These interventions achieved dramatic reductions in malaria, particularly in the first year of the trial in lower transmission areas. Despite an approximate 10-fold reduction in malaria infections, this was not sufficient to end malaria in this setting during the trial period.

In the lower transmission strata, many HFCAs had very few RDT-positive infections. What is the next step for these communities? The NMEP is now looking to implement SIP plus MDA both in the remaining priority transmission areas from the trial site as well as to expand this to other districts and provinces based on a stratification framework.^11^ In a growing number of HFCAs in Southern Province, the total number of confirmed cases is sufficiently small that the health workers can proceed with a case investigation into the household and the neighborhood of an index case. This approach is also under further study to determine the most efficient approaches of finding and clearing additional infections through household and potentially neighboring household assessments or treatments.^12^ In some settings, an examination of the capacity and work within the entire HFCA may be relevant to explore the extent to which some households or neighborhoods may be responsible for repeated cases, the role of even more localized travel in fueling transmission, and the opportunities to include additional specific interventions (e.g., reactive IRS, larviciding, and reactive drug treatments) to target remaining transmission.

Evaluating the impact of malaria interventions on reducing transmission and malaria health outcomes can be challenging. In this trial, investigators used both RDT and PCR testing and four approaches that helped measure intervention outcomes. These included evaluating differences in infection incidence from a longitudinal cohort, confirmed case reporting by health facilities and their linked CHWs, parasite prevalence from surveys conducted during the peak transmission season, and parasite genetic profiles. No single metric would have been both robust and an accurate measure of all the changes. However, because the effect estimates were similar across the measures, we gained additional confidence in the direction and magnitude of the observed outcomes. Measuring intervention effects becomes challenging when transmission is very low and only a few infections are observed; infections and clinical cases become more difficult to find, and it is challenging to interpret exactly what intervention stopped them from occurring. That said, measurements of specific effects on mosquitoes, parasites, and people will remain critical to our understanding of next steps in progress to end malaria.

The trial results are both informative and encouraging for the next steps in malaria transmission reduction on the road to elimination. The current Zambia standard package of interventions is similar to the recommendations across endemic countries in sub-Saharan Africa. Quality administration of the standard intervention package with modest enhancements may markedly and rapidly reduce malaria and suggests that this package could, in some settings, reset malaria epidemiology over a relatively short time frame. Although malaria elimination was not achieved during the trial period, the findings here suggest three next steps.

1. A first priority for the WHO-proposed “high-burden high-impact” response^13^ and for the countries in sub-Saharan Africa and their partners may be to support a locally adapted and tailored program strategy that likely includes very high coverage and use of LLINs, high coverage and expanded targeting of IRS with a highly effective insecticide, expanded access to diagnosis and case management at the community level, logistic/supply chain improvement to avoid stock-outs for RDTs and ACTs, improved case reporting, and community engagement. Additional interventions (e.g., seasonal malaria chemoprevention or improved case management in the private sector) could also be prioritized depending on the local context. Further evaluations should be conducted to examine the effects of such a scale-up.
2. Vectorial capacity remains high in many places in Africa even with vector control interventions focused on indoor biting and resting and requires development of new tools such as attractive toxic sugar baits, for example, that might address outdoor transmission. Insecticide resistance, as well as changing mosquito behavior and species composition, will present challenges to efforts against malaria and must be overcome. Next-generation insecticides for IRS and LLINs, including the synergist piperonyl butoxide for LLINs, will be essential in staying one step ahead of the ever-evolving vector.
3. The proactive use of drugs in populations has an important place in transmission reduction (not just in burden reduction such as the use of seasonal malaria chemoprevention). The use of drugs includes both improving access to treating symptomatic malaria cases and clearing infections in the overall population through mass treatment strategies. Further exploration of preventive strategies with drugs merits attention across the spectrum of transmission intensity and seasonality. Given that many of the trial sites achieved very low case rates, drug use to clear those last cases and stop transmission focally will become a critical step. At present, existing drugs are useful for many of these strategies; however, in the future, new drugs will be needed.

The randomized controlled trial described in this supplement informs these next steps. It showed that quality delivery of the standard package of interventions as recommended by the WHO for malaria-endemic countries in sub-Saharan Africa, along with carefully targeted and implemented population-wide drug-based strategies in lower transmission settings, may significantly add to reductions in malaria transmission and, in some areas, may allow national programs to start aiming for subnational malaria elimination.
